# Genome Sequence of the Necrotrophic Plant Pathogen *Alternaria brassicicola* Abra43

**DOI:** 10.1128/genomeA.01559-17

**Published:** 2018-02-08

**Authors:** Elodie Belmas, Martial Briand, Anthony Kwasiborski, Justine Colou, Guillaume N’Guyen, Béatrice Iacomi, Philippe Grappin, Claire Campion, Philippe Simoneau, Matthieu Barret, Thomas Guillemette

**Affiliations:** a IRHS, INRA, AGROCAMPUS-Ouest, Université d’Angers, SFR 4207 QUASAV, Beaucouzé, France; b Universitatea de Științe Agronomice și Medicina Veterinara București, Bucharest, Romania

## Abstract

*Alternaria brassicicola* causes dark spot (or black spot) disease, which is one of the most common and destructive fungal diseases of *Brassicaceae* spp. worldwide. Here, we report the draft genome sequence of strain Abra43. The assembly comprises 29 scaffolds, with an *N*_50_ value of 2.1 Mb. The assembled genome was 31,036,461 bp in length, with a G+C content of 50.85%.

## GENOME ANNOUNCEMENT

The fungal *Alternaria* genus belongs to the class *Dothideomycetes* and includes many saprophytic and pathogenic species (1). This genus contains many destructive plant pathogens (2), and diseases caused by *Alternaria* spp. are common on many crops, ornamentals, and weeds. *Alternaria brassicicola* causes black spot disease on a wide range of *Brassicaceae* spp., including many economically important oilseed, vegetable, condiment, and fodder crop species, and is routinely used as a model necrotrophic pathogen in studies with *Arabidopsis thaliana* (2). The pathogen can infect all aerial parts of the plant, including pods, seeds, and stems. The typical symptoms correspond to black necrotic lesions, often surrounded by chlorotic areas. *A. brassicicola* is notably the dominating *Alternaria* sp. in *Brassica* sp. seed crops and may be responsible for high yield losses (3–5). As necrotrophic pathogens, which actively kill host tissue as they colonize and thrive on the contents of dead cells, *Alternaria* spp. utilize a variety of pathogenicity factors throughout the infection process. For instance, they secrete an arsenal of host cell wall-degrading enzymes and diverse secondary metabolites, especially toxins, required for plant penetration and nutrient consumption (6, 7). The Abra43 strain was isolated in 1999 from radish seeds during quality controls of French commercial lots. Then, Abra43 was purified by monospore isolation and identified as *A. brassicicola* based on morphological and molecular analyses (8). This strain exhibits a strong aggressiveness on cabbage and *Arabidopsis* sp. leaves and is also efficiently transmitted to seeds (9, 10).

Genomic DNA was extracted from mycelium from a 7-day-old colony growing on potato dextrose agar at 24°C from procedures described in reference 11. The genome sequence of Abra43 was obtained through a combination of PacBio RS II single-molecule real-time (SMRT) technology (Icahn Institute for Genomics and Multiscale Biology, NY, USA) and the HiSeq4000 platform (BGI Tech Solution, Hong Kong). Long reads obtained with 5 SMRT cells were initially assembled with HGAP3 (12), and the assembly was corrected with 150 paired-end reads through Pilon (13). The draft genome of *A. brassicicola* Abra43 consisted of 29 contigs with an *N*_50_ value of 2.1 Mb. The total length of the assembled genome was 31,036,461 bp, with a G+C content of 50.85%.

The *A. brassicicola* Abra43 genome was estimated to have 12,456 protein-coding genes based on the EuGene software (14). The only other public genomic sequence of *A. brassicicola* (for the ATCC 96836 strain) contained 10,514 predicted genes, and the proportion of gaps between scaffolding boards in this sequence was estimated to be 5.2% (1). This new draft genome will support further investigations related to pathogenicity and fungal transmission to seeds.

### Accession number(s).

The draft genome sequence of *A. brassicicola* Abra43 has been deposited in GenBank under accession no. PHFN00000000. The version described in this article is the first version, PHFN01000000.
